# Genome-Wide Association Analysis and Candidate Gene Prediction of Wheat Wet Gluten Content

**DOI:** 10.3390/ijms27020827

**Published:** 2026-01-14

**Authors:** Congcong Liu, Lei Zeng, Cong Wang, Linlin Jia, Wenxu Li, Ziju Dai, Maomao Qin, Jinna Hou, Zhensheng Lei, Zhengfu Zhou

**Affiliations:** 1 Henan Institute of Crop Molecular Breeding, Henan Academy of Agricultural Sciences, Zhengzhou 450001, China; liucc226@163.com (C.L.); zenglei@163.com (L.Z.); wangcong12042022@163.com (C.W.); linlinjia0513@163.com (L.J.); tinbingye@163.com (W.L.); zijudai@163.com (Z.D.); qmm1988630@126.com (M.Q.); houjinna_1982@163.com (J.H.); 2 Shennong Laboratory, Zhengzhou 450002, China

**Keywords:** wheat, wet gluten content, genome-wide association study, candidate gene, molecular marker

## Abstract

The wet gluten content (WGC) of wheat is a key indicator of wheat-processing quality, and its genetic basis is extremely critical in breeding. This study evaluated the WGC of 207 wheat accessions under three growing seasons from a natural population. Nine quantitative trait loci (QTLs) explained 7.61–15.18% of phenotypic variation in a genome-wide association study (GWAS) using a 660K SNP array. Among them, *qWGC6B.2* on chromosome 6BL was consistently detected across multiple environments, accounting for 10.08–12.27% of variation. Incorporating grain transcriptome data led to the identification of *TaWGC6B.1* (*TraesCS6B02G386700*), which is highly expressed in developing endosperm and strongly correlated with WGC. A competitive allele specific PCR (KASP) marker development and validation indicated that the *Whaas68366_GG* allele significantly enhanced gene expression and WGC. This study identified key genes and molecular markers, providing theoretical and technical support for WGC genetic improvement in wheat (*Triticum aestivum* L.).

## 1. Introduction

Around 17% of the world’s cultivated grain area is planted with wheat (*Triticum aestivum* L.), making it one of the most important staple crops [1]. Wheat flour plays an essential role in food production, and is used for baking bread, biscuits, cakes, noodles, steamed buns, and more [2]. Wheat production directly impacts the food industry and national food security. WGC is a critical criterion in wheat grading and is lower in Chinese varieties of strong-gluten wheat than in their foreign counterparts. For strong wheat, medium-strong wheat, medium wheat, and weak gluten wheat, the WGC standard is >30.5%, >28.5%, >24.0%, and <24.0%, respectively, at 14% moisture [3].

Wheat-processing quality is primarily determined by gluten proteins, including glutenins and gliadins, whose structure and ratio dictate wet gluten’s elasticity and extensibility [4]. Bread, buns, and noodles benefit from strong gluten because it enhances dough strength, water absorption, and tensile resistance. Selecting appropriate flour based on WGC can enhance product quality since wheat with different WGC levels is suitable for different end-use products. WGC is significantly correlated with bread volume and overall quality score, but excessive WGC may negatively impact bread firmness. A WGC of 30% is required for optimal bread quality [4,5,6].

Additionally, WGC significantly affects the sensory and palatability characteristics of steamed foods [2]. A uniform gluten network promotes gas retention and fermentation, which eventually results in a softer and elastic texture in steamed buns. The WGC of high-quality steamed buns normally needs to be between 25% and 30% [7]. Noodles made from flour with a higher WGC will be stronger with greater tensile strength but will also be softer and less elastic. Premium noodles usually have a WGC between 28% and 35% [8]. Studies show that WGC between 28% and 32% yields optimal results for volume, color, skin smoothness, internal texture, and flavor in northern Chinese wheat-based foods [9].

Recent years have seen the widespread use of marker-assisted selection (MAS) for breeding wheat varieties with high yield, resistance, quality, and adaptability [10,11]. By using a variety of mapping populations, multiple QTLs associated with WGC have been identified across nearly all chromosomes [12,13,14,15]. Li et al. [16] mapped 15 QTLs on chromosome 5A in an RIL population, with *QWgc.WY-5A.2* explaining the largest proportion of phenotypic variation (28.37–36.76%). A variation explanation rate of 16.43% was reported for *QWgc.sdau-6D* on chromosome 6D by Sun et al. [17]. Li et al. [15] detected 16 QTLs across the 11 chromosomes, with stable loci on 3A, 4D, 5B, and 7B. However, QTL mapping using traditional bi-parental populations has limited resolution and detects only loci segregating between the parents. A genome-wide association study (GWAS) is more precise in detecting QTLs for complex traits by leveraging broader natural genetic variation [18,19,20]. Lou et al. [12] identified nine QTLs for WGC by GWAS on chromosomes 1A, 1B, 3A, 3D, and 6A. Chen et al. [14] found 129 significant SNPs on 2DL, 3AL, 6AS, etc., with explanation rates ranging from 10.4% to 33.3%. Among them, *BS00046964_51* and *IAAV5188* exhibited opposing regulatory effects on WGC on chromosome 6AS.

This study utilized 207 wheat accessions with diverse genetic backgrounds and geographical origins, 224,706 SNPs, and WGC data across three years. A mixed linear model (MLM) was used for GWAS to uncover stable loci, identify candidate genes, and develop KASP marker. The findings aim to provide a theoretical basis for quality wheat breeding and WGC regulatory mechanism research.

## 2. Results

### 2.1. Phenotypic Statistical Analysis of Wheat Wet Gluten Content

**Figure 1:**
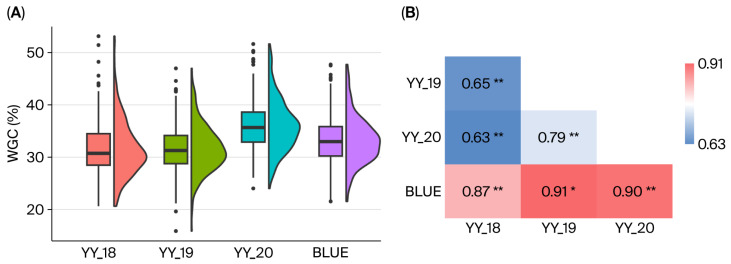
Phenotypic distribution and correlation analysis of wet gluten content (WGC) in the natural population for three different years (YY_18, YY_19 and YY_20) and BLUE values. (**A**) Phenotype distribution of WGC in YY_18, YY_19, YY_20 and BLUE values. (**B**) Pearson correlation coefficient among different years (E1 and E2). YY_18 was planted in Yuanyang County, Henan Province, from 2017 to 2018, YY_19 was planted in Yuanyang County from 2018 to 2019, YY_20 was planted in Yuanyang County from 2019 to 2020, and BLUE represents the Best Linear Unbiased Estimator. * represents *p* < 0.05, ** represents *p* < 0.01.

Wet gluten content (WGC) was measured in a natural wheat population across three environments (growing seasons from 2017 to 2020). The results indicated substantial phenotypic variation in WGC across the different varieties (Figure 1, Table S1). The mean WGC varied from 31.65% to 36.03% in the environments YY_18, YY_19, YY_20 and Best Linear Unbiased Estimator (BLUE) values, with coefficients of variation between 13.69% and 17.08% (Table S2). BLUE values ranged from 21.52% to 47.68%, with an average of 33.23%. Highly significant positive correlations were observed for WGC across all environments, with correlation coefficients ranging from 0.63 to 0.91 (*p* < 0.001). The broad-sense heritability for WGC estimated was 0.91. The results indicated that WGC is predominantly controlled by genetic factors and is a typical polygenic quantitative trait.

### 2.2. Genome-Wide Association Analysis of WGC

**Figure 2:**
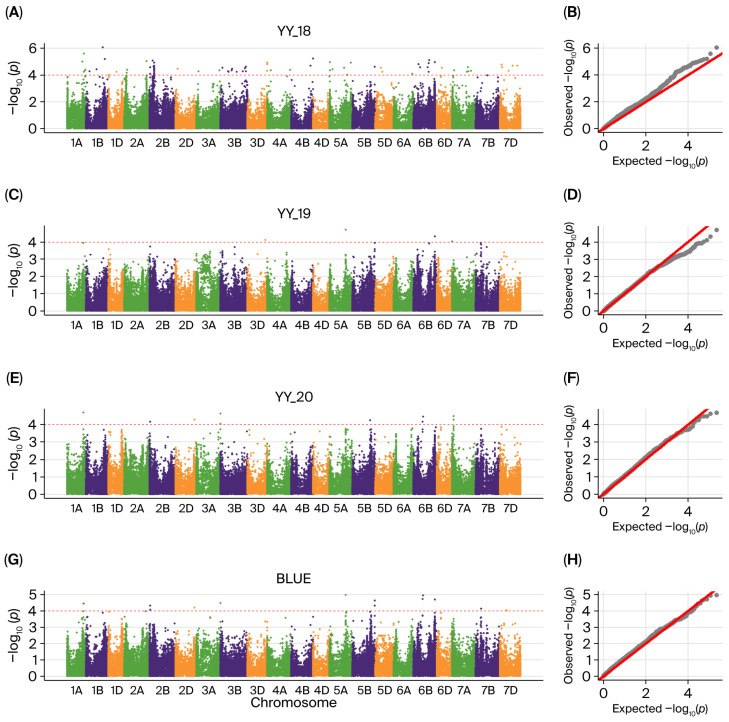
Manhattan plot and Q-Q plot for WGC in different environments. (**A**,**C**,**E**,**G**) represent Manhattan plot for WGC in YY_18, YY_19, YY_20 and BLUE, respectively; (**B**,**D**,**F**,**H**) represent Q-Q plot for WGC in YY_18, YY_19, YY_20 and BLUE, respectively. Red horizontal dotted line indicates significance threshold line (−log_10_(*p*) = 4).

Nine quantitative trait loci (QTLs) were identified on chromosomes 1AL, 1BL, 1DS, 5AL, 5BL, 6BS, and 6BL and were stably expressed in at least two environments (Figure 2, Table 1 and Table S3). Additionally, 7.61% to 15.18% of phenotypic variation was explained by these QTLs. Both *qWGC1A.1* and *qWGC1A.2* were found on 1AL, explaining 9.76–10.99% and 10.10–14.02% of variation, respectively. The highest explained variance was seen in *qWGC1B.1* on 1BL (9.28–15.18%). *qWGC1D.1* on 1DS was located at 0.34 Mb and detected in environments YY_18 and YY_19. The *qWGC5A.1* explained 10.92–10.96% of the variance on 5AL, whereas *qWGC5B.1* explained 7.68–8.21% and 9.47–11.30% of the variance on 5BL, respectively. *qWGC6B.1* has been identified on 6BS (10.43–11.10%). *qWGC6B.2* showed stable major-effect QTL characteristics across all environments, with an explained variance of 10.08–12.27%. Different allelic genotypes within each QTL region showed significant differences in WGC, with favorable alleles increasing it by approximately 9.41% to 36.49% (Table S4).

### 2.3. Candidate Gene Analysis

**Figure 3:**
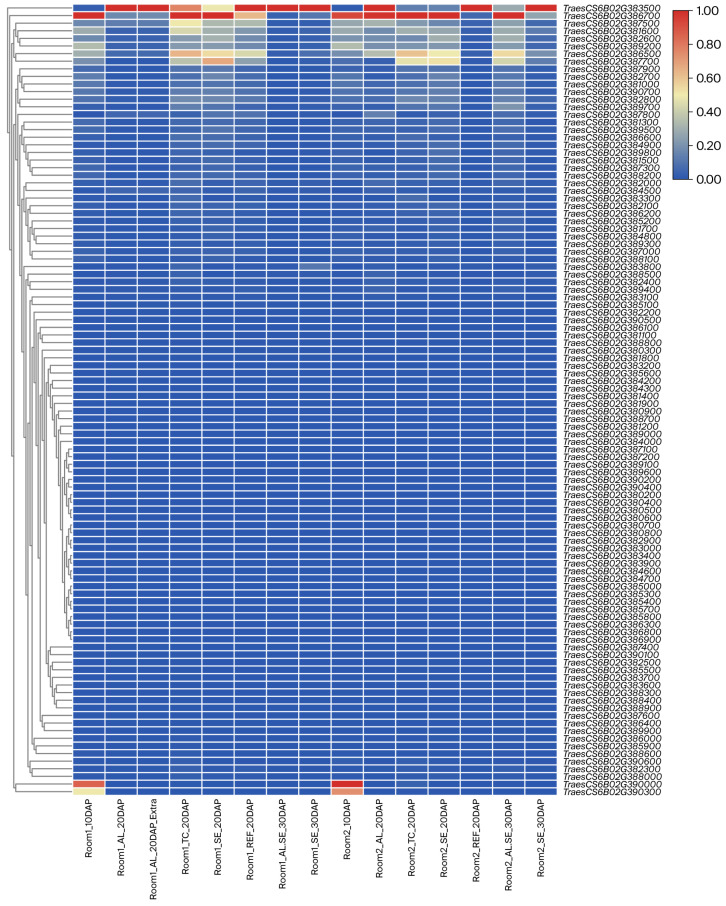
Distribution of gene expression levels within the *qWGC6B.2* by the public database in different wheat tissues. DAP: Days after pollination, AL: Aleurone, TC: Transfer cells, SE: Starchy endosperm, AL.SE: Aleurone tissue to contain contamination from SE cells.

A total of 104 high-confidence genes were annotated in the *qWGC6B.2* interval (Table S5) using the sequence and annotation of the Chinese Spring reference genome. Using the WheatOmics expression database, gene expression patterns were analyzed in various tissues (including the aleurone layer, the transfer cells, and the starchy endosperm) during various grain development stages (10DAP, 20DAP, 30DAP). *TraesCS6B02G383500* and *TraesCS6B02G386700* were identified as highly expressed genes (Figure 3).

*TraesCS6B02G386700* was consistently highly expressed across all tissues and time points, and was especially predominantly expressed in the endosperm, whereas *TraesCS6B02G383500* exhibited higher activity in the aleurone layer and transfer cells. Specifically, the expression level of *TraesCS6B02G386700* was significantly higher than that of *TraesCS6B02G383500* in SE_10DAP and SE_20DAP. However, its expression decreased in SE_30DAP.

**Figure 4:**
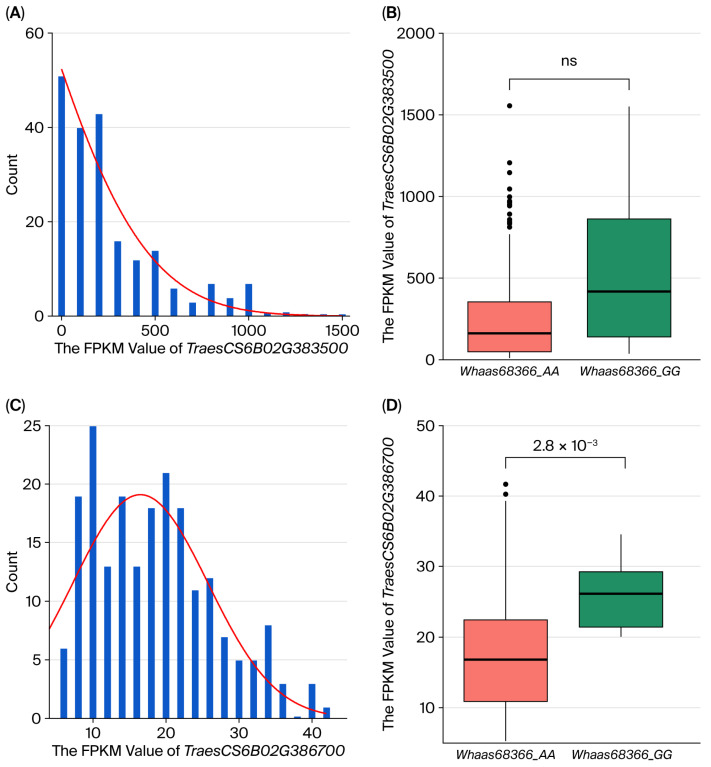
Distribution and comparative analysis with different alleles of the fragments per kilobase Million (FPKM) value of *TraesCS6B02G383500* and *TraesCS6B02G386700.* (**A**,**C**) Distribution of *TraesCS6B02G383500* and *TraesCS6B02G386700* expression levels in the natural population, respectively. (**B**,**D**) The expression levels of *TraesCS6B02G383500* and *TraesCS6B02G386700* between the varieties with *Whaas68366*. The genotypes of *Whaas68366_AA* and *Whaas68366_GG* correspond to the two homozygous allelic variants detected by *Whaas68366* in wheat varieties in natural population. ns: no significance.

The expression of *TraesCS6B02G386700* was further validated by RNA-seq data from grains 20 days after pollination (FPKM: 5.28–41.68) in the natural population, and was significantly lower than *TraesCS6B02G383500* (10.85–1555.87) (Table S6). It was 25.99 in the *Whaas68366_GG* genotype compared to 17.88 in the *Whaas68366_AA* genotype, reflecting a 45.34% increase (Figure 4D). These results support the designation of *TraesCS6B02G386700* as the candidate gene for *TaWGC6B.1*.

Analysis of two key genes in developing grains at 20 days after pollination within a natural population revealed that *TraesCS6B02G383500* expression (FPKM) exhibited extensive variation across accessions, ranging from 10.85 to 1555.87. In contrast, *TraesCS6B02G386700* maintained relatively stable expression levels (5.28–41.68 FPKM). Subsequent allelic genotyping at the *Whaas68366* locus demonstrated no significant divergence in *TraesCS6B02G383500* expression among genotypes (*p* > 0.05), whereas *TraesCS6B02G386700* displayed highly significant genotype-dependent differences (*p* < 0.01).

### 2.4. eGWAS and Expression QTL Mapping of TaWGC6B.1

**Figure 5:**
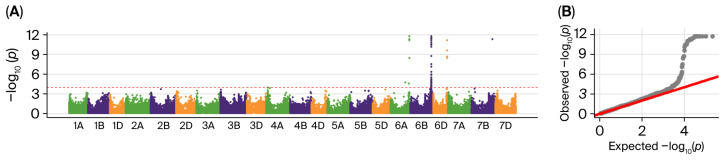
Manhattan plot and Q-Q plot for the expression levels of *TaWGC6B.1*. (**A**) Manhattan plot, (**B**) Q-Q plot. Red horizontal dotted line indicates significance threshold line (−log_10_(*p*) = 4).

Using 660K SNP array genotypes and *TaWGC6B.1* expression levels, expression GWAS (eGWAS) identified five expression QTLs (eQTLs) (Figure 5, Table 2). Two *trans*-eQTLs on 6AL (*qWGCe6A.1*, *qWGCe6A.2*) contributed 11.12% and 10.76–27.74% of variation, one *trans*-eQTL on 6BL (*qWGCe6B.1*) explained 7.66–28.24.4%, and one on 6DL (*qWGCe6D.1*) explained 20.88–25.78% and one on 7BL (*qWGCe7B.1*, 26.07%).

Interestingly, the expression-regulating SNP *Whaas58965* of *TaWGC6B.1* and the WGC-related SNP *Whaas68366* were located in the same linkage block, separated by only 642,068 bases, suggesting that WGC could be controlled by *TaWGC6B.1*.

### 2.5. KASP Marker Development and Validation

A KASP marker was developed based on the *Whaas68366* SNP within *qWGC6B.2* (Table S7). A total of 8 accessions were *Whaas68366_GG* and 186 were *Whaas68366_AA*. As compared to the *Whaas68366_AA* genotype, the *Whaas68366_GG* genotype showed significantly higher WGC in all environments, increasing by 32.70%, 27.52%, 24.76%, and 28.18%, respectively (Figure S1, Table S4).

**Figure 6:**
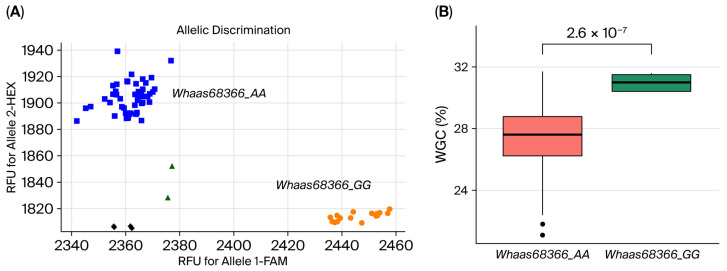
KASP marker and comparative analysis of WGC in wheat varieties with different allele genotypes of *Whaas68366* in population II. (**A**) KASP assay for *Whaas68366* showing *Whaas68366_GG* on FAM and *Whaas68366_AA* on HEX clusters. (**B**) Comparative analysis of WGC in wheat varieties with *Whaas68366_AA* and *Whaas68366_GG* in population II. The orange circle, blue square and green triangle represent the genotype of *Whaas68366_GG*, *Whaas68366_AA* and heterozygous of *Whaas68366*, respectively. The black square represents the control without DNA template.

Further validation in 107 lines from Population II identified 102 *Whaas68366_AA* genotypes and 5 *Whaas68366_GG* genotypes (Figure 6, Table S8). *Whaas68366_GG* lines had an average WGC of 30.98%, which was significantly higher than *Whaas68366_AA* lines (27.39%), which indicated an increase of 13.11%. These results demonstrate that *Whaas68366_GG* is a favorable allele and can be used for marker-assisted selection in breeding high-WGC wheat varieties.

## 3. Discussion

Accurate determination of wet gluten content is essential for quality assessment and breeding, as it directly influences water absorption, dough viscoelasticity, and product formability [21,22]. Instrument-based measurements were taken in this study across multiple environments and BLUE values ranged from 21.52 to 47.68%, indicating a wide range of genetic diversity. By selective breeding, significant potential can be gained for improving wheat quality based on the substantial genetic diversity in wet gluten content observed in this study.

Nine WGC-associated QTLs were identified on chromosomes 1AL (two loci), 1BL, 1DS, 5AL, 5BL (two loci), 6BS, and 6BL using GWAS. Among them, *qWGC1A.1*, *qWGC1D.1*, and *qWGC5A.1* co-localized with previously reported QTLs *QWgc.his-1A* (535 Mb), *QWgc.his-1D* (23 Mb), and *QWgc.his-5A* (540 Mb) [23], suggesting that these regions possess stable genetic determinants. As a result of the overlap with previously reported QTLs related to wheat gluten content and composition, these findings are robust and emphasize the importance of this chromosomal region. The identification of these QTLs offers valuable insights into breeding programs aimed at enhancing wheat quality. Additionally, the complex genetic interactions among QTLs and the polygenic nature of gluten content may complicate marker-assisted selection, requiring further research.

Wheat glutenin and gliadin content reflects the ability of wheat proteins to absorb water and form networks [24]. Zhou et al. [25] identified 33 QTLs related to glutenin components in recombinant inbred lines, including *QAx1.1AS-1*, *QBy.1BL-1*, *QLMW.1DS-1*, and *QLMW.5AL-1*, which co-localize with *qWGC1A.2*, *qWGC1B.1*, *qWGC1D.1*, and *qWGC5A.1* in this study. *qWGC1D.1* was also co-localized with *1DS-1* controlling α-gliadin and gliadin accumulation in RIL population by Zhou et al. [26]. Additionally, *Qωg5B.4* (578 Mb) and *Qωg5B.5* (690 Mb) were found on 5BL to influence ω- and γ-gliadin expression, respectively. *Qωg6B.3* (323 Mb) on 6BS and *Qγg6B.2* (659 Mb) on 6BL also affect gliadin subunit expression [27].

This study identified *qWGC6B.2* on 6BL as a stable major-effect QTL, with its favorable allele increasing WGC by 24.76–32.70%. *TaWGC6B.1* (*TraesCS6B02G386700*), a cytochrome B-c1 complex subunit gene, has been identified as a potential candidate gene for *qWGC6B.2*. Mitochondrial cytochrome complexes play crucial roles in cellular respiration and energy production [28,29,30]. As developing grains are metabolically active and energy-intensive, the elevated expression of *TaWGC6B.1* in the endosperm suggests that it may regulate ATP-dependent processes such as protein synthesis and starch accumulation. These physiological functions directly impact gluten protein content and grain quality. Moreover, expression QTL (eQTL) analysis revealed that *TaWGC6B.1* plays both *cis*- and *trans*-acting roles in regulating its expression. The colocalization of the WGC phenotype-associated SNP (*Whaas68366*) with an eQTL SNP for *TaWGC6B.1* (*Whaas58965*) within the same linkage block strengthens the hypothesis that WGC is modulated by transcriptional regulation of this gene. Understanding the transcriptional regulation of key genes like *TaWGC6B.1* enables breeders to develop crops with enhanced traits through targeted manipulation of gene expression pathways.

Another candidate gene, *TraesCS6B02G383500*, belonging to the LEA (late embryogenesis abundant) D-II family, was identified in addition to *TaWGC6B.1*. The role of dehydrins in abiotic stress tolerance, particularly under drought and heat conditions, is well-known [31,32,33,34]. As grain filling and protein accumulation are highly sensitive to environmental factors, the expression of dehydrins could indirectly affect WGC by stabilizing storage protein synthesis. It is consistent with findings in rice [35] and barley [33], where LEA proteins were implicated in seed vigor, desiccation tolerance, and seed quality.

The integration of transcriptomic, genomic, and phenotypic data in this study exemplifies the power of multi-omics approaches in unraveling complex agronomic traits. To uncover regulatory mechanisms beyond transcript abundance, future studies could include epigenetic profiling (e.g., DNA methylation and histone modification) and proteomic analyses. According to Zhou et al. [36], some genes regulate WGC via transcriptional or epigenetic mechanisms, influencing gluten protein ratios through promoter DNA methylation. A premature stop codon has been found in *TraesCS1D02G009900* in region *1DS.1*, altering the HMW-GS/LMW-GS ratio and enhancing gluten aggregation, as reported by Zhou et al. [25]. Together, these findings indicate that gluten protein composition and proportional regulation are important determinants of wet gluten content (WGC), and that further study of underlying transcriptional and epigenetic mechanisms is warranted.

The successful development and validation of a competitive allele specific PCR (KASP) marker, *Whaas68366*, provides a practical application of the genetic findings. In addition to effectively distinguishing high-WGC genotypes, this marker demonstrated utility across multiple environments and populations. In breeding programs that aim to select high-quality wheat varieties, such markers represent an effective tool for genomic selection and precision breeding.

## 4. Materials and Methods

### 4.1. Plant Material and Cultivation

This study used 207 accessions from Henan, Jiangsu, Shaanxi, Sichuan, and Yunnan provinces in China, along with six other countries, including landraces, cultivars, and elite breeding lines [37]. A secondary population (Population II), comprising 107 wheat varieties from the southern region of Huang-Huai, was constructed to provide validation data.

Natural populations were grown at the Modern Agricultural Research and Development Base in Yuanyang County (113°97′ E, 35°5′ N), Henan Province, during 2017–2018, 2018–2019, and 2019–2020 growing seasons. Population II was grown at the same location in 2019–2020. The average temperature is 10.12 °C (http://www.tianqihoubao.com/lishi/hnyuanyang.html, accessed on 18 June 2025) and the average annual precipitation is 410.76 mm (https://www.tianqi24.com/historycity/, accessed on 18 June 2025) in the wheat-growing season. The varieties were planted in two rows, each two meters long, with 20 cm row spacing and 10 cm plant spacing, with two replicates. Field management followed local standard agronomic practices. Mature grains were harvested and air-dried.

### 4.2. Phenotypic Evaluation and Data Analysis

Moisture content was determined using the IM9500 multifunctional near-infrared analyzer (Perten, Stockholm, Sweden). SKCS 4100 single-kernel characterization system was used to measure the hardness index of 300 grains randomly selected from each sample, following GB/T 21304-2007 [38]. The soaking water content was adjusted based on the hardness index: 14% for soft wheat (HI < 40), 15% for mixed wheat (40 < HI < 60), and 16% for coarse wheat (HI > 60), with a soaking duration of 16–18 h [39].

The flour was prepared using a Bühler laboratory mill (Bühler, Uzwil, Swiss Confederation) according to American Association of Cereal Chemists (AACC) Method 26-20 [40], with an extraction rate of 70%. Wet gluten content (WGC) was measured with a GM2200 gluten washer (Perten, Stockholm, Sweden) following AACC Method 38-12A [41]. Each sample was measured twice replicates.

Descriptive statistics and correlation analyses of WGC across environments were conducted using IBM SPSS Statistics 22 (IBM, Chicago, IL, USA). Broad-sense heritability (*H*^2^) was obtained using the R software version 3.5.3 (R Development Core Team, 2019) package lme4 vision 1.1-14 (https://cran.r-project.org/src/contrib/Archive/lme4/, accessed on 20 December 2020) [42]. The best linear unbiased Estimator (BLUE) was also calculated using the lem4 software in R and used for the GWAS.

### 4.3. 660K SNP Genotyping and Genome-Wide Association Study (GWAS)

High-quality genomic DNA was extracted from the leaves at the seedling stage of 207 wheat varieties in natural population using the CTAB method. The quality and concentration of DNA were detected by NanoDrop2000 spectrophotometer (Thermo Scientific, Waltham, MA, USA) and 1.2% (*v*/*v*) agarose gel electrophoresis. Illumina wheat 660K SNP array (CapitalBio Technology Inc., Beijing, China) was used to genotype genomic DNA. BeadStudio software (https://illumina-beadstudio.software.informer.com/) (Illumina Inc., San Diego, CA, USA) was used to process the raw data. All SNPs with missing rates > 10%, minor allele frequencies < 5%, or ambiguous genotypes were excluded. SNPs of high quality were retained for further analyses of population structure and kinship matrix [37].

The population structure was conducted using the STRUCTURE software version 2.3.4 (Pritchard Lab, Stanford University, San Francisco, CA, USA) with a model-based Bayesian cluster analysis. The number of ancestral clusters (K) was varied from two to seven to explore potential population subdivisions. For each K, 1000 burn-in periods and 1000 Markov-Chain replicates were conducted. The adhoc statistic ΔK, derived from the rate of change in LnP(K), was used to determine the true subpopulations. The kinship matrix was generated with TASSEL 5.0 and was incorporated as a random-effect factor in GWAS.

GWAS was conducted using TASSEL 5.0 using a mixed linear model (MLM), accounting for population structure (Q) and kinship matrix (K). SNPs with *p*-values < 1.0 × 10^−4^ were considered significant. The Manhattan plot and Q-Q plot were generated using the R software version 3.5.3 (R Development Core Team, 2019) package CMplot version 4.5.1 (https://cran.r-project.org/web/packages/CMplot/index.html, accessed on 24 May 2024). As a result of each QTL, the peak SNP that explained the highest proportion of phenotypic variance was identified, and its corresponding 5 Mb region was defined as the QTL interval. The ggplot2 version 3.4.0 (https://cran.r-project.org/src/contrib/Archive/ggplot2/, accessed on 26 May 2024) was used to analyze and visualize genotypic differences.

### 4.4. Candidate Gene Prediction and Analysis

The IWGSC RefSeq v1.1 reference genome (https://www.wheatgenome.org) was used to identify gene candidates within the *qWGC6B.2* region. Expression Browser (http://www.wheat-expression.com) was used to analyze gene expression profiles between grain development stages (10, 20, 30 days after pollination) and tissues (AL, TC, SE, REF, and AL.SE) [43].

An analysis of RNA-seq data from 20 DAP grains of the natural population [42] was performed to identify significantly expressed genes, with a focus on stable and highly expressed genes in the endosperm.

### 4.5. Competitive Allele Specific PCR (KASP) Models and Their Application

KASP marker was designed using peak SNP sequence from the polymarker website (http://www.polymarker.info/) [37]. The PCR reaction volume was prepared according to the KASP assay protocol: 5 μL KASP Master Mix, 1.4 μL Primer Mix containing F1, F2, R12, and ddH_2_O in a ratio of 12:12:30:46, 0.08 μL MgCl_2_, 1 μL of genomic DNA (100 ng/μL), and 2.52 μL ddH_2_O, making up a total volume of 10 μL. The designed KASP marker was subsequently used to genotype the wheat varieties in population II on the CFX Connect™ Real-Time System. PCR-cycling conditions included an initial denaturation step at 95 °C for 15 min; 10 cycles, denaturation at 95 °C for 20 s, annealing at 64 °C for 60 s, gradually reduce for 1 °C each cycle; 35 cycles, denaturation at 95 °C for 20 s, annealing at 57 °C for 60 s; 37 °C for 1 min, the signal was read after 1 min at 37 °C.

The alleles associated with higher WGC were considered favorable. Marker-assisted selection (MAS) was performed on accessions with favorable alleles.

## 5. Conclusions

Nine QTLs associated with wet gluten content were identified in a natural wheat population, with *qWGC6B.2* on chromosome 6BL being a stable major-effect locus. Expression levels of the candidate gene *TaWGC6B.1* correlated significantly with wet gluten content. The developed KASP marker *Whaas68366* effectively distinguished favorable alleles and significantly enhanced WGC, proving its potential in molecular breeding. These findings provide key loci, candidate genes, and practical markers for wheat quality improvement.

## Figures and Tables

**Table 1:** Distribution of significant SNP of wet gluten content (WGC) in different environments.

Num	QTL	Peak SNP	Chr	Pos (Mb)	−log_10_ (*p*)	R^2^ (%)	Environment
1	*qWGC1A.1*	*Whaas33494*	1AL	516.7	4.23–4.68	9.76–10.99	YY_20, BLUE
2	*qWGC1A.2*	*Whaas05721*	1AL	532.8	4.36–5.58	10.10–14.02	YY_18, BLUE
3	*qWGC1B.1*	*Whaas46733*	1BL	515.5	4.04–6.03	9.28–15.18	YY_18, BLUE
4	*qWGC1D.1*	*Whaas08068*	1DS	0.3	4.01–4.17	7.61–9.80	YY_18, YY_19
5	*qWGC5A.1*	*Whaas93836*	5AL	510.1	4.69–4.74	10.92–10.96	YY_19, BLUE
6	*qWGC5B.1*	*Whaas41968*	5BL	555.1	4.07–4.26	7.68–8.21	YY_20, BLUE
7	*qWGC5B.2*	*Whaas43373*	5BL	693.0	4.09–4.57	9.47–11.30	YY_18, BLUE
8	*qWGC6B.1*	*Whaas33025*	6BS	301.0	4.45–4.79	10.43–11.10	YY_20, BLUE
9	*qWGC6B.2*	*Whaas68366*	6BL	659.2	4.31–4.94	10.08–12.27	YY_18, YY_19, BLUE

**Table 2:** Information for QTL associated with the expression levels of *TaWGC6B.1*.

Num	QTL	Peak SNP	Chr	Pos (Mb)	−log_10_ (*p)*	R^2^
1	*qWGCe6A.1*	*Whaas86516*	6A	454.7	4.71	11.12
2	*qWGCe6A.2*	*Whaas49643*	6A	585.4	4.56–11.77	10.76–27.74
3	*qWGCe6B.1*	*Whaas58965*	6B	659.8	4.00–11.78	7.66–28.24
4	*qWGCe6D.1*	*Whaas29943*	6D	437.8	8.40–11.15	20.88–25.78
5	*QWGCe7B.1*	*Whaas58461*	7B	658.2	11.25	26.07

## Data Availability

The original contributions presented in this study are included in the article and Supplementary Materials. Further inquiries can be directed to the corresponding authors.

## Supplementary Materials

The following supporting information can be downloaded at https://www.mdpi.com/article/10.3390/ijms27020827/s1.

## Abbreviations

The following abbreviations are used in this manuscript:

WGC	Wet gluten content
MLM	Mixed liner model
GWAS	Genome-wide association study
BLUE	Best linear unbiased estimate
QTL	Quantitative trait locus
SNP	Single-nucleotide polymorphism
Q-Q	Quantile–quantile
PVE	Phenotypic variation explained
IWGSC	International Wheat Genome Sequence Consortium
SE	Starchy endosperm
AL	Aleurone
TC	Transfer cells
W	The whole endosperm
AL.SE	Aleurone tissue to contain contamination from SE cells
DAP	Days after pollination
KASP	Competitive Allele Specific PCR
