# Supplementary material for: Genome-Wide Association Analysis and Candidate Gene Prediction of Wheat Wet Gluten Content
Source: Int J Mol Sci. 2026 Jan 14;27(2):827. doi: 10.3390/ijms27020827 (PMC12841151; doi:10.3390/ijms27020827)
Supplement: Supplementary file 1 [file ijms-27-00827-s001.zip › Supplyment Figure.pdf]

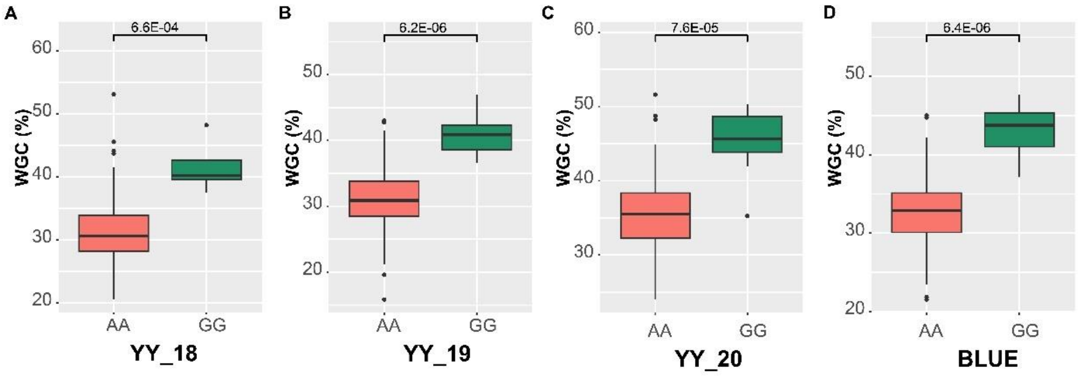

Figure S1. Comparative analysis of WGC in wheat varieties with different allele genotype of *Whaas68366* in natural population. * YY_18: 2017 to 2018, YY_19: 2018 to 2019, YY_20: 2019 to 2020.
